# Integrating intramuscular fat radiomics with hamstrings-to-quadriceps structure and function ratios to predict future hamstring strain injury

**DOI:** 10.1371/journal.pdig.0001144

**Published:** 2025-12-23

**Authors:** Akanksha Sharma, Daniel R. Smith, Alexis B. Slutsky-Ganesh, Jed A. Diekfuss, Jennifer A. Hogg, Kim D. Barber Foss, Christopher D. Riehm, Augustin C. Ogier, Constance P. Michel, David Bendahan, Richard Danilkowicz, Joseph Lamplot, Destin Hill, Kyle Hammond, Charles Kenyon, Gregory D. Myer, Anant Madabhushi

**Affiliations:** 1 Wallace H. Coulter Department of Biomedical Engineering, Georgia Institute of Technology & Emory University, Atlanta, Georgia, United States of America; 2 Emory Sports Performance And Research Center (SPARC), Flowery Branch, Georgia, United States of America; 3 Emory Sports Medicine Center, Atlanta, Georgia, United States of America; 4 Department of Orthopedics, Emory University School of Medicine, Atlanta, Georgia, United States of America; 5 Department of Kinesiology, University of North Carolina at Greensboro, Greensboro, North Carolina, United States of America; 6 Department of Veterans Affairs, Atlanta VA Medical Center, Decatur, Georgia, United States of America; 7 Department of Health and Human Performance, University of Tennessee at Chattanooga, Chattanooga, Tennessee, United States of America; 8 Department of Diagnostic and Interventional Radiology, Lausanne University Hospital and University of Lausanne, Lausanne, Switzerland; 9 Aix-Marseille Université, CNRS, CRMBM, Marseille, France; 10 Endeavor Health, Chicago, Illinois, United States of America; 11 Department of Rehabilitation Medicine, University of Washington, Seattle, Washington, United States of America; 12 The Micheli Center for Sports Injury Prevention, Waltham, Massachusetts, United States of America; 13 Youth Physical Development Centre, Cardiff Metropolitan University, Wales, United Kingdom; Instituto Politécnico Nacional Escuela Superior de Medicina: Instituto Politecnico Nacional Escuela Superior de Medicina, MEXICO

## Abstract

We performed a prospective, longitudinal investigation to determine whether magnetic resonance imaging (MRI)-based radiomic features from thigh intramuscular fat (IMF) can predict future hamstring strain injury (HSI). Further, we sought to determine if muscle imbalance or injury profile along with radiomics could increase prediction accuracy. This study analyzed IDEAL MRI scans of 93 professional American football players (9 injured, 84 uninjured). Radiomic features relating to textural patterns of IMF were extracted from bilateral hamstring and quadriceps muscles. Feature selection identified non-correlated features that were more strongly associated with future HSI. The K-nearest neighbor classifier was employed to assess the performance of the following models: radiomics of hamstring IMF ${\;(M}_{r}^{H})$ and quadriceps IMF ${\;(M}_{r}^{Q}),\;$ muscle imbalance features (*M*_*b*_) and injury profile features (*M*_*i*_), as also integrated models for *M*_*r*_, *M*_*b*_ and $M_{i}\;(M_{r+b+i})$, and integrated *M*_*r*_ and *M*_*b*_ (*M*_*r+b*_) where $\;M_{r\;}\epsilon \;\left\{{\;M}_{r}^{H}{\;,M}_{r}^{Q}\right\}.$ $M_{r+b+i}^{H}$ (area under the curve (AUC)=0.79; 95%CI:0.78-0.79) significantly outperformed $M_{r+b+i}^{Q}$ (AUC = 0.69; 95% CI: 0.68-0.70), $M_{r+b}^{H}$ (AUC = 0.74; 95% CI: 0.73–0.75), $M_{r}^{H}$ (AUC = 0.68; 95% CI: 0.67–0.69), *M*_*i*_ (AUC = 0.68; 95% CI: 0.68-0.69) as well as *M*_*b*_ (AUC = 0.64; 95% CI: 0.63-0.65). The results indicate that future HSI can be predicted when incorporating radiomics features from hamstrings IMF with muscle imbalance and injury profile data. These novel findings merit further validation in a larger population, one that includes populations of injured and uninjured participants, a limitation acknowledged in current study. This approach could inform future strategies to identify factors to mitigate the risk of HSI not just in elite male athletes but also in athletes of both sexes and any level of participation.

## Author summary

We explored whether MRI scans of thigh muscles, specifically looking at fat within the muscles, known as intramuscular fat, could help us predict future hamstring injuries in professional football players. We focused on detailed texture features of the scans, called radiomic patterns, found in the hamstrings and quadriceps, and combined this data with information about past injuries and muscle imbalances. We analyzed MRI scans from 93 players and used machine learning models to determine which combinations of data were most accurate. We found that radiomic features from the hamstrings, when combined with muscle imbalance and injury history, gave us the best prediction of which players were at risk of future injury. Our findings suggest that this information could be used to identify players at higher risk of hamstring injuries before they occur. This may lead to more personalized injury prevention strategies, though we believe larger studies are needed to confirm and expand on these results.

## Introduction

Hamstring strain injury (HSI) is most frequently associated with sports requiring sprinting or sudden starts and stops, including soccer, football, basketball, and tennis. HSI constitutes 12%–15% of all injuries in different sports [1]. While a high percentage of players who sustain HSI can return to sport, some never achieve their prior level of function or performance or performance level status [2]. Despite extensive research on HSI prevention and management, HSIs continue to cause significant time lost from athletic competition. Time lost in return to play can extend from weeks to months and is correlated with injury severity [1,3]. Injury severity is also correlated with reinjury rates, which range from 12%–41% and result in negative outcomes for both the athlete’s athletic career and quality of life [1].

HSI is common at all levels of sport, although the effects are arguably most impactful at the professional level. In professional soccer, HSI averages 3 per team and accounts for greater than 8 missed matches; with an economic impact of €11,373,179 (12,433,955 USD) [4]. In the American National Football League (NFL) alone, HSI is the second most frequent preseason injury, occurring at a rate of 1.79 per 1000 athlete exposures during practices and 4.07 per 1000 athlete exposures in games [5]. Between 1998–2007, the NFL reported 2.2 HSIs per 1000 athlete exposures in training camps [5]. These injuries burden teams with substantial financial and performance loss, highlighting the need for predictive modeling to help reduce potential occurrences.

Following HSI, a combination of factors, including traumatic and chronic injuries, often results in increased levels of intramuscular fat (IMF) [6], which encompasses multiple types of adipose tissues present below the muscle fascia. During the complex healing process of the injuries, the skeletal muscle fibers are often replaced by fatty and fibrous tissues [7]. This replacement, known as fatty infiltration, results in disruption of the tissue’s function, as the IMF compromises the contractile components of skeletal muscle. Additionally, these muscular injuries can also result in tissue scarring and fibrous tissue replacement of skeletal muscle fibers. The replacement of these muscle fibers and infiltration of fibrous and fatty tissues causes significant decreases in muscle flexibility and contractile function [8,9] leading to an increased risk of subsequent injuries. However, it should be noted that musculoskeletal injuries are not the only factors leading to increased levels of intramuscular fat (IMF), including genetic predispositions genetic predispositions [10], hormonal imbalance [10] or metabolic disorders like obesity [11,12] all which can alter relative IMF.

Decreased contractile function of the hamstrings following injury creates an imbalance in hamstring muscle strength, a significant risk factor in HSI injury [13]. This imbalance has traditionally been measured in two ways: bilateral hamstring strength asymmetry and hamstring-to-quadriceps (HQ) strength ratio. However, hamstring muscle strength imbalance does not require traumatic injury to be present and increases the risk of HSI. While several studies have aimed to support hamstring strength imbalance as a risk factor for HSI, not all studies agree, indicating that measuring this imbalance is only a partial contributor to future HSI [14–16]. Another potential muscle imbalance that increases the risk of HSI is the difference in muscle size between the hamstrings and quadricep muscles, due to its correlation with muscle strength [17].

The clinical Hamstring Outcome Score (HaOS) [18] is a tool to identify prior hamstring injuries and quantify a qualitative assessment of the perceived severity of previous hamstring injuries. HaOS characterizes soreness, pain, activities (sports), and quality of life, and is analogous to commonly used scores such as Hip And Groin Outcome Score (HAGOS) [19], Foot and Ankle Outcome Scores (FAOS) [20], and Knee Osteoarthritis Outcome Score (KOOS) [21]. A prior study suggested that HaOS outcomes are associated with previous and future HSI and can stratify players at risk of new injuries when combined with HSI history [18]. Prior lower extremity injury symptoms may provide additive information potentially vital to identify risk for future injury or even assess HSI severity that would inform safe return to sport.

The imaging modalities, such as ultrasound and MRI, are often used to evaluate the nature and severity of the injury. Ultrasound has high sensitivity to diagnose these injuries, but only when the assessment is performed immediately following injury and conducted by a skilled technician [22]. Thus, MRI is traditionally the preferred diagnostic tool to evaluate deeper muscle injuries while also discriminating between new injuries or scars from prior injury [3,23]. However, quantitative-based MR techniques (T2-weighted imaging, diffusion-weighted imaging) have shown promise for differentiating muscle microstructure differences in athletes acutely following HSI [24], and have the unique potential to estimate timelines for return-to-sport following HSI [25]. However, current approaches have not robustly identified MR-derived signatures at the time of first injury or upon return to play that predict re-injury risk. Thus, its use in predicting the risk of future HSI remains uncertain. Research is still underway to predict the risk of future HSI. An exhaustive review [26] concluded that there is a lack of available evidence about the association of MRI-derived signatures at the time of injury or return-to-play to predict re-injury risk. However, moderate evidence suggests that intratendinous injuries found in MRI scans at the time of injury are associated with a high re-injury risk [26]. High levels of IMF are indicative of serious injury [27]. During HSI, high levels of IMF are retained in muscles [6] due to fatty infiltration, resulting in disruption of muscle tissue function [28].

MRI radiomics provides the potential to provide further insights into these MRI-derived signatures. Radiomics is a term that refers to the computational extraction of multiple quantitative features from medical images (computed tomography [29], MRI [30]). These features could describe texture, shape, intensity, statistical distribution, and other attributes of diseased regions, offering valuable insights to inform diagnosis, treatment planning, prognosis, and personalized medicine [31]. A recent study used radiomics of multiparametric MRI to identify HSI and return-to-play duration using machine learning [25]. However, the study used MRI scans at the time of injury (≤ 7 days from injury) on a relatively small cohort of 32 players. Furthermore, only one radiomics-based study exists in the literature, and its feature analysis was limited to the hamstring muscles alone. This narrow focus may not adequately capture the comprehensive functional status of the lower extremity that is relevant to future hamstring strain injury (HSI) risk [25].

In summary, previous studies [18,25] have not included prospective scans and limited the inclusion of radiomics of quadriceps muscles as well as other variables to predict future HSI. In this study, we performed a prospective longitudinal investigation to determine whether machine learning informed MRI-based radiomic features from hamstring and quadriceps IMF can predict future HSI. In addition, we sought to determine if muscle imbalance (HQ ratio of cross-sectional area (CSA) of muscles and torque generation) or injury profile (self-reported symptoms and injury history) could predict future HSI. Finally, we sought to isolate the top-performing model (s) via the exploration of different combinations of radiomics features, muscle imbalance, and injury profiles. To the best of our knowledge, this study is novel in its attempt to integrate radiomics with muscle imbalance and injury profile information.

The objectives of this study were to (a) evaluate the association of radiomics from hamstring and quadriceps muscle’s intramuscular fat with the occurrence of future HSI using prospective MRI-derived metrics; (b) to investigate whether the integration of injury profile variables, namely HaOS and prior HSI, muscle morphology and strength imbalance, with radiomics, increased the ability to predict future HSI.

## Materials and methods

**Fig 1:**
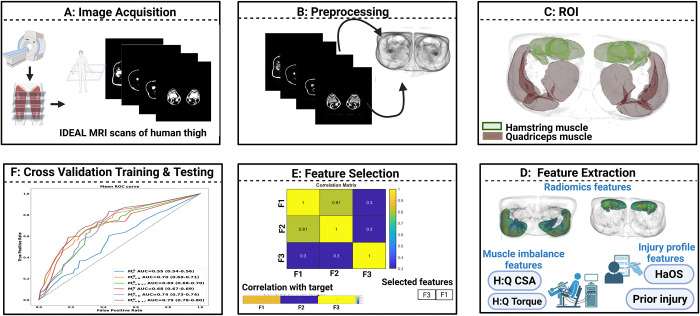
Block diagram for the system workflow. **A)** IDEAL MRI scans of right and left thighs, scanned from the knee. **B)** Scans were normalized by removing the bias field using low-pass filtering. **C)** The quadriceps muscle and hamstring muscle’s region of interest (ROI) was delineated using a semi-automated algorithm. **D)** Radiomics features from the ROIs were extracted at the voxel level. Muscle imbalance variables, the HQ ratio for CSA and the HQ ratio for torque, were computed. Injury profile variables, namely prior injury history and total Hamstring Outcome Score (HaOS) score, were used for model construction. **E)** Feature selection was performed to identify non-correlated features that were more strongly associated with future HSI. **F)** Model training and evaluation using 250 iterations of three-fold cross-validation on K-nearest neighbor classifier. Results were computed using area under the curve (AUC), its confidence interval, sensitivity, specificity, and accuracy.

The radiomics methodology included data acquisition, preprocessing, formation of a region of interest, radiomics feature extraction, feature selection, and cross-validation using the KNN classifier (Fig 1).

### Study population

The current investigation included a prospective cohort of professional American football players. The investigation was approved by the Institutional Review Board at Emory University (STUDY00003840), with data collection taking place at Emory Sports Performance and Research Center (SPARC)and all participants provided written informed consent prior to participation. All procedures were performed in accordance with the Declaration of Helsinki and relevant guidelines. Clinical trial number: not applicable. A total of 112 athletes enrolled in this study (mean age: 25.35 ± 2.37 years; mean height: 183.16 ± 7.21 cm; mean mass: 96.58 ± 19.31 kg). Inclusion criteria were male professional athletes actively engaged in the competitive season, 18 years or older, able to provide written consent, and no contraindications to MRI. Athletes not medically cleared to participate in sport, were unable to provide written consent, or had contraindications to MRI were excluded. Of the 112 total enrolled athletes, 19 were excluded from the present analyses for the following reasons: did not complete MRI testing (n = 7), muscle masks not quality checked prior to data query (n = 6; data query performed in December of 2023), poor muscle mask data quality (n = 3), missing one stack of IDEAL sequence data (see below for MR acquisition/stacking; n = 2), and HaOS data not collected (n = 1), resulting in 93 participants with complete datasets (i.e., had useable MRI/masks, muscle imbalance outcomes, and injury profiling data). Three stacks of IDEAL scans were collected, starting approximately from the iliac crest, and ending mid-patella. The three stacks shared overlapping slices and were bound into one image. Imaging parameters for individual IDEAL scans were: field of view = 450 × 450 × 200 mm; resolution = 1.76 × 1.76 m; slice thickness = 5mm; spacing = 0mm; acquisition matrix = 256 × 256; reconstructed matrix = 512 × 512; slices = 40; TR/TE = 8.54 ms/3.94 ms. The IDEAL sequence of MRI was used because it separates the water and fat signals within the body, better facilitating visualization and differentiation of tissue’s fat levels from tissue water in muscles and, organs. Of the 93 athletes included in the final analysis, nine participants (prior HSI = 5) developed an in-season HSI [referred to as injured (*HS*^+^)], and 84 participants (prior HSI = 17) had no in-season HSI [referred to as uninjured (*HS*^−^)]. Table 1 presents summary statistics of both groups.

**Table 1:** Sample characteristics.

	Uninjured (*HS*^−^)	Injured (*HS*^+^)	p-value
**Count (n)**	84	9	
**Age (µ ± σ), years**	25.34 ± 2.38	23.77 ± 2.16	0.0739
**Weight (µ ± σ), kg**	95.44 ± 18.00	89.10 ± 13.41	0.3390
**Height (µ ± σ), cm**	183.4 ± 7.3	182.2 ± 6.9	0.5986
**Prior injury, yes | no**	17 yes | 67 no	5 yes | 4 no	
**Injured limb side**	--	3 left | 6 right	
**HaOS, (µ ± σ)**	94.12 ± 6.70	95.78 ± 2.43	0.7403
**CSA HM (µ ± σ),**	16229.01 ± 2384.29	15392.88 ± 2545.90	0.1317
**CSA QM (µ ± σ),**	23696.66 ± 3165.11	23721.55 ± 4047.23	0.9480
**A_H:Q_ (µ ± σ),**	0.68 ± 0.07	0.65 ± 0.04	0.4056
**T_H:Q_ (µ ± σ),**	0.49 ± 0.10	0.39 ± 0.09	0.0060
**Right Quad Torque (µ ± σ)**	142.24 ± 27.95	145.50 ± 26.21	0.8250
**Left Quad Torque(µ ± σ)**	137.47 ± 27.21	144.25 ± 28.40	0.5200
**Right Hamstring Torque (µ ± σ)**	69.49 ± 19.02	54.07 ± 16.09	0.0214
**Left Hamstring Torque(µ ± σ)**	66.93 ± 18.45	56.64 ± 11.67	0.0419

Note: HM-Hamstring muscle, QM-Quadriceps muscle, µ-mean, σ-standard deviation, HaOS-Hamstring Outcome Score, CSA- Cross section area, T_H:Q_ – torque. P-value stated using Wilcoxon ranksum test.

### Preprocessing

MRI images are often prone to nonuniformity in intensities that vary with pulse sequence, field strength, and body tissues [32]. These intensity nonuniformities can affect image interpretation and radiomic feature extraction [33]. Data were preprocessed by an established bias field correction method [32] which involves performing a low-pass Gaussian filtering. The Gaussian filter provides an estimation of the bias field, which is then subtracted from the original MRI scans, resulting in more uniform image intensity distributions [33].

### Region of interest (ROI)

Hamstrings and quadriceps muscle ROIs were delineated by a semi-automated segmentation technique using the IDEAL water-contrast images [34]. This method requires manual segmentation of at least two slices for each muscle (most proximal and most distal ends) which are propagated using a combination of diffeomorphic registrations to create a full 3D muscle mask. Though two slices are required, adding more slices improves the propagation robustness and reduces the volume error with 9 slices showing robust segmentation [35]. Nine slices were manually segmented throughout the four hamstring muscles (biceps femoris short and long heads, semitendinosus, and semimembranosus) and three quadriceps muscles (vastus lateralis, vastus medialis, and rectus femoris) to inform semi-automated segmentation resulting in a full muscle mask for each muscle. Resultant muscle masks were checked for accuracy and edited if needed by an expert (D.R.S). The 3D volume mask of the hamstring and quadriceps muscles are illustrated in the S1 and S2 Files.

### Radiomic feature extraction

Radiomic features were extracted in 3D from the IDEAL fat-contrast images using the masks of corresponding muscle groups generated from the IDEAL water-contrast images with the Pyradiomics library [36]. Features derived corresponded to shape, first-order statistics, gray-level co-occurrence matrix, gray-level size zone matrix, gray-level run length matrix, and neighboring gray-tone difference matrix descriptors. For each ROI we obtained a 107-dimensional feature vector. Detailed descriptions of radiomics features are summarized in S3 File.

### Computation of muscle imbalance variables

Each muscle’s CSA was calculated at the widest part of the muscle for the individual hamstring and quadriceps muscles and then combined to calculate a total CSA for the hamstring and quadricep muscle groups. CSA of the hamstring, CSA of the quadriceps, and the ratio of HQ CSA averaged across limbs, referred to as $A_{H:Q},$ were used for analysis. To assess the overall thigh muscle function and torque production, each participant completed an isokinetic dynamometer testing protocol (Biodex System 4 Pro, Biodex Medical Systems Inc., Shirley, NY). Each participant performed one set of 10 repetitions of isokinetic knee extension and flexion (concentric/concentric) at 180°/s from a seated position at a 90° hip angle. The torque data were low-pass filtered at 100Hz. Discrete kinetic variables were exported from the dynamometer software. The HQ peak torque ratio, referred as $T_{H:Q}\;$was calculated as the absolute peak knee flexion torque across all repetitions divided by the absolute knee extension torque across all repetitions and then averaged across limbs.

### Injury profile variables

All participants completed the HaOS [37] survey, which consists of two parts. Part 1 asks about hamstring injury history (yes/no) and if yes, time since most recent injury and duration of injury. Part 2 consists of nineteen questions clustered within 45 dimensions relative to current levels of soreness, pain, function/activity, and quality of life assessment within the past week. Each question is scored from 0 to 4, from no complaints to maximum complaints, with each side assessed individually. The total HaOS composite score was used.

### Feature selection and classification

Features were z-normalized [38] resulting in zero mean and unit variance throughout all training samples. Non-correlated features were identified that were strongly associated with the future HSI using the Spearman correlation coefficient of 0.6. Minimum redundancy and maximum relevance [39] was used to select the best five features after the removal of correlated features. Multiple models were constructed corresponding to a) radiomics features: hamstring IMF$\;\left(M_{r}^{H}\right)$, quadriceps IMF $\left(M_{r}^{Q}\right)$, combined hamstring and quadriceps IMF$\;\left(M_{r}^{H+Q}\right)$; b) muscle imbalance features: HQ ratio of CSA ($A_{H:Q}$), HQ ratio of torque ($T_{H:Q})$, combined muscle imbalance features ${(M}_{b})$; c) combined radiomics and muscle imbalance features $\left(M_{r+b}^{H}{\;,\;M}_{r+b}^{Q}\;,\;M_{r+b}^{H+Q}\right)$; d) injury profile features: previous HSI (*P*_*i*_), HaOS and combined previous HSI and HaOS score (*M*_*i*_); e) combined radiomics and injury profile features $\left(M_{r+i}^{H}{\;,\;M}_{r+i}^{Q}\;,\;M_{r+i}^{H+Q}\right)$; f) combined radiomics, muscle imbalance, and injury profile features $\left(M_{r+b+i}^{H}{\;,\;M}_{r+b+i}^{Q}\;,\;M_{r+b+i}^{H+Q}\right)$. Results were computed with five classifiers, namely K-nearest neighbor (KNN), Logistic Regression (LR), Random Forest (RF), Support Vector Machine (SVM) (with radial basis function(rbf) and linear kernel). The feature vectors were concatenated when integrating different models. Optimal features in post-classification analysis were identified as those with a > 20% maximum frequency of occurrence in 250 x three-fold cross-validation.

### Statistical analysis

Classification models were trained and tested using 250 iterations of three-fold cross-validation. Holdout testing could not be performed due to the small event rate. For each iteration, participant indices for the fold were selected randomly in the beginning and were fixed throughout the study. Participants within a fold were not repeated in other folds. Samples from the minority class were repeated to balance the data. Sensitivity, specificity, accuracy, area under the curve (AUC), and 95% confidence interval (CI) of AUC were computed to assess cross-validation performance. Sensitivity refers to the percentage of correctly classified injured participants; specificity refers to the percentage of correctly classified uninjured participants. Python’s Scikit library was used for implementation [40]. Wilcoxon rank-sum test was used to determine statistically significant differences in the performance of the two models using the statannotation library [41]. Statistical improvement was noted if the mean AUC of model A was more than model B and the AUC of cross-validation for model A and B were significantly different using the Wilcoxon rank-sum test.

### Unsupervised clustering analysis

An unsupervised analysis was performed to assess the efficacy of radiomics features before and after combination with injury profile and muscle imbalance features. A random training set of 5 injured and 69 uninjured participants and a test set, comprising 4 injured and 15 uninjured participants was formed. The training set was used to find uncorrelated features closely associated with future HSI. Uniform Manifold Approximation and Projection (UMAP) embeddings of selected features in the test set were plotted for models $M_{r}^{H}$, $M_{r+b}^{H}$, $M_{r+b+i}^{H}$, respectively. In addition, we also performed a hierarchical clustering on the test set using an average linkage and Euclidean distance.

## Results

### Sample characteristics

This study included 84 *HS*^−^ participants (prior HSI = 17) and 9 *HS*^+^ participants (prior HSI = 5). Table 1 presents summary statistics of both groups. Among all the characteristics, $T_{H:Q}$, left hamstring torque and right hamstring torque significantly differed between the groups.

### Future injury prediction using radiomics features

**Fig 2:**
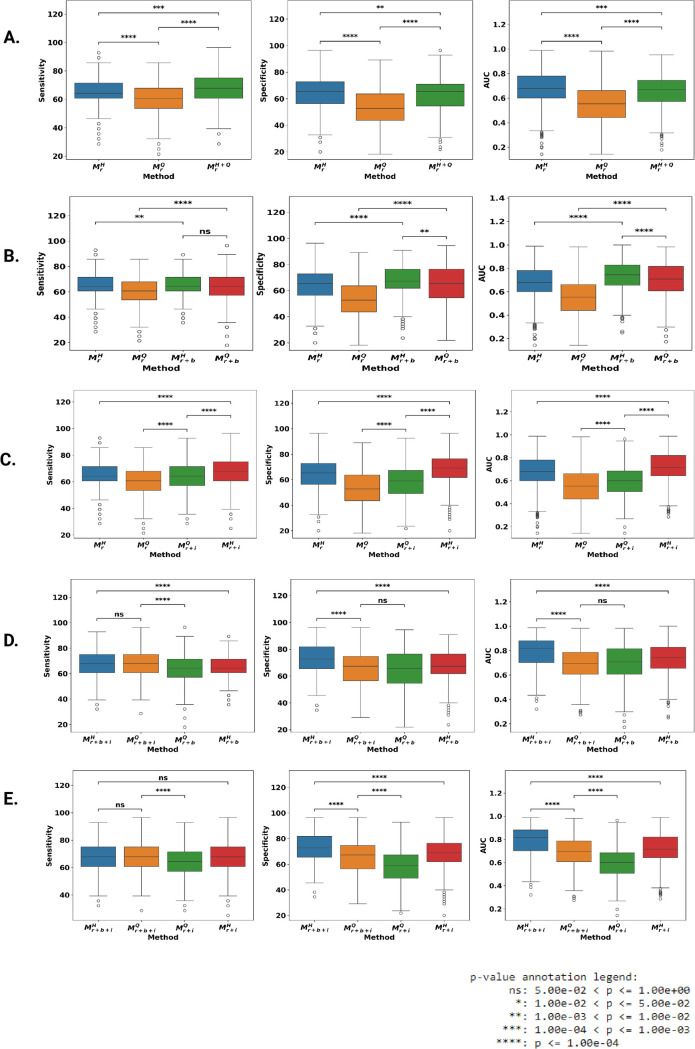
Box plot of the performance metrics (sensitivity, specificity and AUC) and their pairwise statistical comparison for different model using Wilcoxon test. **A)** $M_{r}^{Q}$, $M_{r}^{H},\;M_{r}^{H+Q}$ B) $M_{r}^{Q}$, $M_{r}^{H}$,$M_{r}^{Q}$, $M_{r+b}^{H}$, $M_{r+b}^{Q}$ C) $M_{r}^{Q}$, $M_{r}^{H}$, $M_{r+i}^{H}$, $M_{r+i}^{Q}$ D) $M_{r+b+i}^{H}$, $M_{r+b+i}^{Q}$, $M_{r+b}^{Q}$, $M_{r+b}^{H}$ E) $M_{r+b+i}^{H}$, $M_{r+b+i}^{Q}$, $M_{r+i}^{Q}$, $M_{r+i}^{H}$.

**Fig 3:**
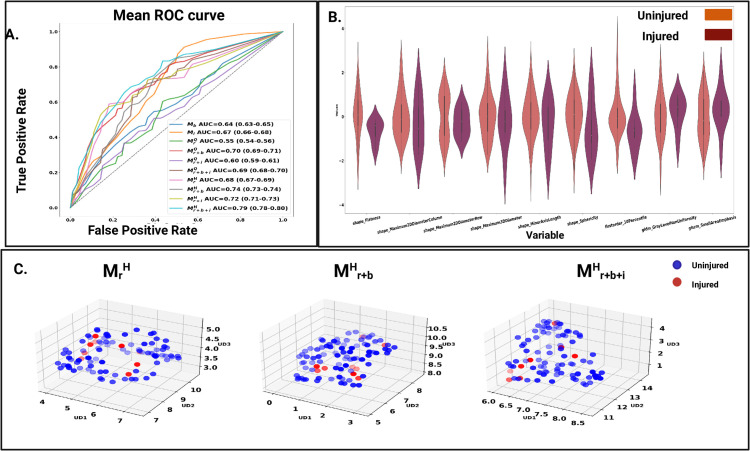
Supervised classification performance. A) ROC plots for cross validation models $M_{r}^{Q},$ $M_{r+b}^{Q},\;M_{r+i}^{Q},$ $M_{r+b+i}^{Q}$, $M_{r}^{H},$ $M_{r+b}^{H},\;M_{r+i}^{H}$ $M_{r+b+i}^{H}$, $M_{b},\;M_{i},\;\;$ B). Violin plot of features selected in cross-validation for $M_{r}^{H}$ C). UMAP embeddings of frequently selected features in supervised classification for cross validation models, $M_{r}^{H},$ $M_{r+b}^{H},$, $M_{r+b+i}^{H}$.

**Fig 4:**
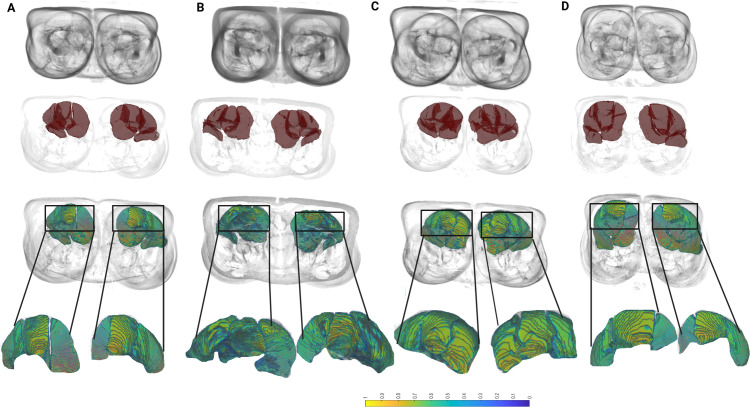
Visualization of one of the frequently selected features from$M_{r}^{H},$ gray-level non-uniformity computed from the gray-level dependence matrix. First row represents MRI images, second row shows hamstring mask overlaid on MRI images, and third row shows feature map. Fourth row represents zoomed representation of feature map for bounded regions in black square. Columns A and B represent two injured participants; columns C and D represent two uninjured participants. Regions outlined in black box show differences in texture feature intensity between uninjured and injured participants. Intensity is lower in injured compared to uninjured participants.

We first assessed the contribution of radiomics features from $M_{r}^{H}$and $M_{r}^{Q}$ models to predict future injury. Mean results are shown in Table 2. The highest AUC of 0.67 (0.67–0.69) was obtained for $M_{r}^{H}$, with sensitivity of 63.87% and specificity of 65.54%. Results for $M_{r}^{H}$ were significantly better than for $M_{r}^{Q}$ (Fig 2A). In the $M_{r}^{H+Q}$ model, the resulting AUC significantly differ from $M_{r}^{Q}$ and $M_{r}^{H}$ (Fig 2A). Fig 3A shows the ROC curve for the individual muscles radiomics model and the different integrated models presented later. Fig 3B shows violin plots of optimal features in $M_{r}^{H}$. The features selected were dominant from shape class, including flatness, maximum 2D diameter column, maximum 2D diameter row, maximum 3D diameter, minor axis length and sphericity. Other optimal features were 10^*th*^ percentile from first-order features, gray-level non-uniformity from the gray-level dependence matrix and small area emphasis from gray level size zone matrix. Fig 4 shows MRI volume, hamstring mask, and corresponding feature map overlaid on MRI volume for two uninjured and two injured participants. The Injured participants had a low scale of feature values on the upper regions of hamstring muscles compared to uninjured participants. The 3D volume of the feature map for the uninjured and injured participants is shown in the S4 and S5 Files, respectively. In addition, we performed an uninjured vs injured limb identification using $M_{r}^{Q}$ and $M_{r}^{H}$ models. Out of 9 injured participants, there were 3 with left limb injured and 6 with right limb injured. A similar cross validation paradigm was followed with 250 × 3 fold. The results using both the models are illustrated below in Table 3. Sensitivity refers to percentage of correctly identified left leg injured and specificity refers to percentage of correctly identified right leg injured subjects.

**Table 2:** Performance evaluation of radiomics, muscle imbalance, and injury profile variables, as well as their integration in terms of accuracy, sensitivity, specificity, AUC, and 95% CI.

Model Group	Model	Sensitivity (%)	Specificity (%)	Accuracy (%)	AUC	95% CI (AUC)
**Radiomics features**	$M_{r}^{H}$	**63.87**	**65.54**	**64.72**	**0.6789**	**0.67-0.69**
	$M_{r}^{Q}$	48.00	59.47	53.84	0.5523	0.54-0.56
**Injury profile features**	$M_{i}$	66.31	56.31	61.22	0.6702	0.66-0.68
**Radiomics and injury profile features**	$M_{r+i}^{H}$	**68.13**	**68.03**	**68.08**	**0.7186**	**0.71-0.72**
	$M_{r+i}^{Q}$	54.71	63.06	58.96	0.5987	0.59-0.61
**Muscle imbalance features**	$M_{b}$	60.58	60.38	60.48	0.6419	0.63-0.65
**Radiomics and** **muscle imbalance features**	$M_{r+b}^{H}$	**71.11**	**64.61**	**67.8**	**0.7358**	**0.73-0.75**
	$M_{r+b}^{Q}$	66.13	64.84	65.47	0.6981	0.69-0.71
**Radiomics, muscle imbalance, and injury profile features**	$M_{r+b+i}^{H}$	**78.44**	**67.89**	**73.07**	**0.7879**	**0.78-0.80**
	$M_{r+b+i}^{Q}$	**63.60**	**67.61**	**65.64**	**0.6932**	**0.68-0.70**

Note: Highest performing model within each model group has been highlighted in bold.

**Table 3:** Performance evaluation of radiomics model for identification of injured vs uninjured limb.

	Sensitivity (%)	Specificity (%)	Accuracy (%)	AUC	95% CI
$M_{r}^{H}$	97.2	25	61.1	0.611	0.60-0.62
$M_{r}^{Q}$	100	20	60	0.603	0.59-0.61

### Future injury prediction using prospective muscle imbalance features

$A_{H:Q}$and $T_{H:Q}\;$were both associated with HSI prediction (AUC = 0.65, 95% CI: 0.64-0.66; AUC = 0.79, 95% CI: 0.79-0.81; respectively). Although $T_{H:Q}$ performed better than $A_{H:Q}$ when combining the variables into $M_{b\;,}$ the model obtained (AUC = 0.64; 95% CI: 0.63-0.65). Results for individual variables are listed in the S6 File (Table 1). The confusion matrix for the best performing model in Table 2 are shown in Fig A in S6 File. The results of top performing model with other classifiers, LR, RF, and SVM (rbf) and SVM (linear) are listed in Table 2 in Appendix S6 File under section A. It was observed that performance of linear classifiers like KNN, LR and SVM (linear) was better than RF and SVM (rbf kernel). Among other radiomics based studies using MRI, study in [42] also used multiple classifier, but SVM stood out. Authors in [25] also used SVM classifier to predict return-to-sport and perform uninjured vs injured limb identification. Upon analysis with different classifiers, we observed KNN consistently provided a better balance between sensitivity and specificity in most of the models. Hence, we preferred it as the final classifier.

### Future injury prediction using combined radiomics and muscle imbalance features

The $M_{r+b}^{H}$ model yielded an AUC of 0.74 (0.73–0.75) while $M_{r+b}^{Q}$ improved the AUC to 0.70 (0.69-0.71) (Table 2). These improvements were significant compared to the individual radiomics model associated with each muscle (Fig 2B). The $M_{r+b}^{H+Q}$ model (S6 File) failed to perform on par with respect to $M_{r+b}^{H}$ alone. The commonly selected radiomics feature in $M_{r+b}^{H}$ and $M_{r}^{H}$ were the same, as shown in Fig 3B.

### Future injury prediction using injury profile features

*M*_*i*_ was evaluated in terms of its association with future HSI, resulting in an AUC of 0.67 (0.66–0.68) with a sensitivity of 66.31% and specificity of 56.31%. However, the classification performance of HaOS alone resulted in an AUC of 0.49 (0.48–0.50), suggesting that HaOS by itself was not associated with HSI, but was bolstered by prior HSI information. The performance of individual injury profile features is shown in the S6 File, Table 1.

### Future injury prediction using combined radiomics and injury profile features

We assessed the contribution of $M_{r}^{H}\;$and $M_{r}^{Q}\;$individually with *M*_*i*_ to predict future injury. Table 2 shows the combined results for each muscle. The highest AUC of 0.72 (0.71–0.72) was obtained for $M_{r+i}^{H}$ and it lead to significant increase in both sensitivity and specificity (Fig 2C). $M_{r+i}^{Q}$ performed significantly better than $M_{r}^{Q}$ with an AUC of 0.60 (0.59–0.61). Frequently selected radiomics features in $M_{r+i}^{H}$ and $M_{r}^{H}$ were the same, as shown in Fig 3B.

### Future hamstring strain injury prediction using fusion of radiomics, muscle imbalance, and injury profile features

The $M_{r+b+i}^{H}$ model improved significantly yielding an AUC of 0.79 (0.78–0.80) while the $M_{r+b+i}^{Q}$ model improved the AUC to 0.69 (0.68–0.70) (Table 2). The improvements were significant for $M_{r+b+i}^{H}$ over previous joint models but $M_{r+b+i}^{Q}$ could see significant improvement only over $M_{r+i}^{Q}\;$ (Fig 2D, 2E). Fig 3C shows the UMAP embeddings of the complete data for the three models, namely $M_{r+b}^{H}$, $M_{r+b+i}^{H}$ and $M_{r}^{H}$. The improvement in integrated models, namely $M_{r+b}^{H}$ and $M_{r+b+i}^{H}$ over $M_{r}^{H}$ is also observed visually, wherein the injured subjects occupy more clustered space as we add *M*_*i*_ and *M*_*b*_ to $M_{r}^{H}$.

### Unsupervised clustering analysis

**Fig 5:**
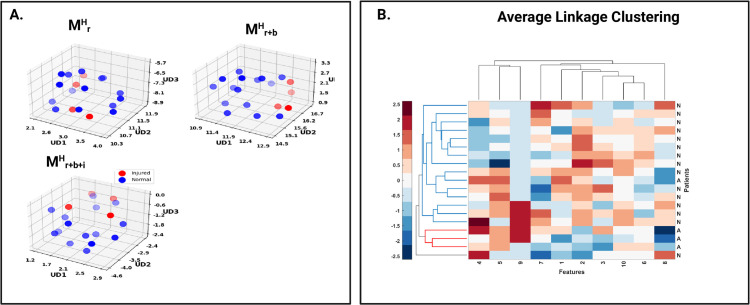
Unsupervised classification performance. A) UMAP embeddings of selected features in holdout test set for the three types of models, $M_{r}^{H},$ $M_{r+b}^{H},$ $M_{r+b+i}^{H}$. Features were selected using a training set. B) Clustermap of same test data using features selected from the training set of $M_{r+b+i}^{H}$.

Fig 5A shows UMAP embeddings of selected features on the test set for each of $M_{r}^{H},\;M_{r+b}^{H},\;M_{r+b+i}^{H}$. The integrated models ($M_{r+b}^{H}$ and $M_{r+b+i}^{H}$ show better separability between 4 injured and 15 uninjured subjects compared to $M_{r}^{H}$. Radiomics features selected from $M_{r}^{H}$ corresponded to the shape-based features including elongation, maximum 2D diameter row, minor axis length, surface volume ratio and texture features such as informational measure of correlation and Inverse Difference Normalized from the gray-level co-occurrence matrix. Fig 5B shows clustering results for $M_{r+b+i}^{H}$. In Euclidian space, data formed two groups corresponding to the uninjured and injured participants.

## Discussion

In this study, we established the utility of combining radiomics features along with injury profile features and muscle imbalance features to predict future HSI. We examined the potential of radiomics features individually, as well as in association with injury profile and muscle imbalance features, to predict HSI. Model $M_{r}^{H}$ had a higher performance relative to $M_{r}^{Q}$ (Fig 3A). The features selected in $M_{r}^{H}$ (Fig 3B) were used to quantify shape, texture, complexity, and homogeneity. The difference in distribution of these features in the fat-contrast IDEAL images suggests that these prospective MRI scans have discriminative IMF signatures associated with future injury and are therefore indicative of changes in IMF levels, suggesting that this change is a potential risk factor for future HSI. We also visualized the the heatmap of gray-level non-uniformity feature computed from the gray-level dependence matrix in the IMF distribution of hamstring muscle in Fig 4. Gray-level non uniformity captures the complexity and variability in the texture of fat distribution of the hamstring muscle. The source for the increased IMF levels in the hamstring muscles could vary between individuals, as IMF contains multiple types of adipose and fatty tissues including intramuscular adipose tissue, or fat that has infiltrated in and between muscle fibers, intramyocellular lipids (IMCL), or liquid droplets which are used as a metabolic fuel source during exercise stored within muscle cells, and fatty tissue between muscle fascicles [43]. As previously discussed, this increase in IMF could be indicative of alteration in tissue composition and fatty infiltration due to prior traumatic or chronic injury, either direct or indirect. This fatty infiltration results in muscles with significantly higher stiffness, decreased muscle fiber contractile length, and decreased force production [8] leading to muscle strength imbalance. These increases are seen further when the IMF is distributed throughout the muscle.

Study by Torres-Velázquez M et al. is the only study to date which used radiomics features from MRI scans of hamstring muscles to predict return-to-play and identify the injured vs uninjured limb from multiple modalities of MRI [25]. The combination of radiomics features from all diffusion tensor imaging and T2-weighted images provided the differentiation between involved and uninvolved limbs with AUC (µ ± σ) of 0.84 ± 0.16 with 14 subjects as left limb injured and 18 subjects with right limb injured. In our study, we have already computed the performance of radiomics features from hamstring muscle to identify injured subjects, yielding an AUC of 0.68 (0.67-0.69). The results for identifying the injured limb (Table 3) show that none of the models adequately identify the right leg injured. A higher count of subjects may boost the predictive power of these models.

The confusion matrices shown Fig A in S6 File highlight that the number of injured participants misclassified as uninjured (referred to here as a false negative (FN) error) consistently decreased when injury profile and muscle imbalance was added to $M_{r}^{H}$. However, only in $M_{r+b}^{H}$, the participants who were uninjured but misclassified as injured (referred to here as false positive (FP) error) were more than those in $M_{r}^{H}$.

However, there are other factors that could affect the predictive effects of the fat signal from the IDEAL MRI scans, particularly in those who have not suffered HSI or other significant injury to the lower limbs. Two of these factors include muscle fiber type and activity level, both of which could correct IMF levels, particularly due to IMCL. For instance, IMCL levels are also subject to change due to activity level, with higher IMCL levels often associated with lower activity, except when accounting for highly trained athletes, particularly those who participate in anaerobic exercise, due to the athlete’s paradox [44]. Alternatively, type I, or slow twitch, muscle fibers, of which quadriceps, in particular, have a broad range of fiber type distribution, ranging from 20% in sprinting athletes to 95% in marathon runners, store significantly higher levels of IMCL than type II, or fast twitch fibers [45]. However, type II fibers are believed to be more at risk for traumatic injuries, such as HSI, due to their explosive nature [46]. It has further been established in the literature that the fat composition of thigh muscles changes due to pathological conditions such as musculoskeletal disorders and metabolic diseases [47].

Type II muscle fibers also play a vital role in explosive movements, such as those measured by the dynamometer and peak torque production measures. Following HSI, within the injured leg, there is muscle loss of both hamstring and quadriceps muscles, whereas in the uninjured leg only the quadriceps significantly atrophy. From a biomechanical muscle structure perspective, prior HSI can alter muscle CSA, indicating muscle atrophy, scar tissue formation, or other alterations due to injury and the healing process. Studies using MRI scans show significant muscle volume loss in the quadriceps and hamstring muscles after injury [48]. In our study, dominance of shape features from hamstring muscles aligns with the aforementioned findings.

Another crucial biomechanical muscle parameter is an individual’s capacity to generate high peak torques using the lower extremity musculature. Excessive dynamic lengthening of an activated muscle beyond its optimum has shown a significant correlation with future injury risk [16]. The muscle fibers tear upon exceeding maximum length, particularly when placed under tension. Additionally, poor muscle balance between the quadriceps and hamstrings can result in abnormal loading mechanics on the muscle fibers and exceed the mechanical limits for the hamstring muscles [49]. The role of the quadriceps muscles with respect to HSI is debatable. Studies [14,16] have previously demonstrated that a low HQ ratio of peak torque was significantly associated with the history of HSI while others stated otherwise [16,50]. Misclassification analysis of injured participants using $M_{r}^{H}\;$highlighted that participants with prior HSI were relatively less frequently misclassified compared to those with no prior HSI. This suggests that subjects with prior HSI appear at a higher risk of future HSI. Subjects with prior HSI might have residual scars or signatures captured by the radiomics features. Study in [51] demonstrated that after injury the muscle goes through a regenerative process with initial scar formation. Further, surrounding muscle undergoes significant atrophy, with an alteration in the viscoelastic properties of the muscle that increases the risk for reinjury [51]. Thus, prior HSI combined with the tendency for the uninjured hamstring muscles to experience atrophy may partially explain the predictive power of our hamstring model.

The potential of MRI measurements to predict the occurrence/recurrence of HSI has demonstrated mixed results in previous studies. Parameters such as maximal percentage transverse area of injury and volume of injury (measures the injury size) correlate with time to return to play and provide the foundation to investigate whether HSI size is related to injury recurrence [51]. Anthropometric measurements, convalescent interval, clinical features, and MRI measurement of an initial HSI also have been evaluated to identify parameters predictive of injury recurrence [51]. Among these, only MRI measurements predict injury recurrence in the subsequent season. Prospective muscle functional MRI scans in conjunction with post-exercise scans have been used to predict HSI and injury recurrence [52]. Changes in signal intensity between scans can detect differences in metabolic characteristics of participants with and without recurrent injuries as well as participants experiencing their first HSI. Our study is unique in that it is the first to identify future HSI using pre-injury MRI scans with a machine learning-informed radiomics approach.

A prior study suggested that HaOS is associated with previous and future HSI and can stratify players at risk of new injuries when combined with HSI history [18]. Our findings using HaOS alone as an associative factor are not in line with other reports [11] (also confirmed by statistical test in Table 1), yet both studies agree that consolidation of prior HSI with HaOS is a better associative factor. Misclassification analysis of injured participants using $M_{i}$ highlighted that participants without a history of prior HSI were more frequently misclassified for their in-season injury risk.

Although review studies [53,54] have yielded inconsistent findings on the influence of HQ torque ratios on the occurrence of future HSI, our findings suggest that $T_{H:Q}$ can be a potential marker of future HSI, with an AUC of 0.79. Previous review studies indicated limited capacity to distinguish between injured and uninjured legs or individuals affected by hamstring strains. However, they did not examine the association between HQ torque ratio and future injury. The recent extensive review study in [55] concluded that there is moderate to strong evidence that the conventional and functional hamstrings to quadriceps strength ratio are not an independent risk factor for hamstring injury. For future injury prediction, the HQ ratio has limited scope. Analysis of strength measures along with other modifiable factors, may better help to understand the association between HQ torque ratio and injury [55]. In our study, the frequently misclassified injured participants using $T_{H:Q}$ had no common trait concerning prior injury. As such, $T_{H:Q}$ may explain additional variance aside from prior HSI, strengthening our model. Our findings suggest that $T_{H:Q}$ can be a potential marker of future HSI, irrespective of prior HSI.

From an additional clinical application perspective, the authors acknowledge that MRI is traditionally considered the gold standard for diagnosing deeper muscle injuries and for distinguishing new injuries from residual scarring of prior injuries [3,23]. However, MRI has rarely been used prospectively, as in the current study, to predict future injury risk. The novel modeling approach presented here represents a key advancement, offering the potential to identify athletes at heightened risk before an injury occurs. This proactive capability opens the door to early intervention strategies that could prevent injuries with profound consequences for an athlete’s season and career. Additionally, the prospective design enhances the utility of MRI by providing a baseline reference that can be compared to post-injury scans, improving the accuracy of determining injury severity. Pre-season baseline scans are already routinely performed in high level athletes, which allows for potential quick application of these modeling approaches to already standard practices. Together, these innovations offer a comprehensive framework: predicting the likelihood of future injuries while guiding clinical decisions when injuries do occur. This dual capability has the potential to transform hamstring injury management, addressing a common challenge that affects athletes at all levels, from youth to elite competition.

We acknowledge that this investigation, despite being one of the largest prospective longitudinal studies involving MR imaging prior to monitored hamstring injury, a limitation to consider with the modeling approach is the limited number of incident injuries in our cohort. Using the robust prospective design, we had limited control over the incident cases of injury. Another prospective study [56] also observed limited HSI in their cohort, 18 HSI and 78 uninjured. While another study [57] indicated an incidence proportion of 5.6%(95%CI: 4.0-7.7) This shows that number of injured subjects on a football team is consistent with prior report. Similar report [25] based on MRI radiomics used only 32 injured participants to examine the association of MRI radiomics with HSI and to predict return-to-play duration. Future studies that have the ability to include more participants in a similar study design would help further validate the reported results.

The best model in our study,${\;M}_{r+b+i}^{H}$ has False Negative Rate (FNR = 21.55%) and False Positive Rate (FPR = 32.11%). While these values are not negligible, both metrics represent substantial improvements compared to the other models evaluated. Misclassifying injured subjects as uninjured (false negatives) poses a significant risk, as these athletes miss the opportunity for early intervention, increasing their likelihood of sustaining a serious injury. Conversely, uninjured subjects misclassified as injured (false positives) may undergo additional interventions unnecessarily. However, these interventions, such as enhanced neuromuscular training or more comprehensive pre-season preparation carry minimal downside and can offer secondary benefits, including reducing the risk of other injuries and improving overall performance. Given this balance, a more sensitive model is preferred, even at the expense of a higher false positive rate, to maximize the opportunity for early detection and prevention of injuries. For reference, Table C in S6 File summarizes the FNR and FPR of the top-performing models.

Nonetheless, future studies should include higher numbers of participants to validate our results. Other dataset-related limitations are the lack of an independent test set and single-site data. Further, we only examined HSI. It would be interesting to determine if we can predict other kinds of sports injuries, such as anterior cruciate ligament injury, using a similar strategy of AI with MRI, muscle imbalance and injury profile fusion modeling. Further, other MRI modalities could be acquired to gain additional information from scans such as DTI, and DWI sequence for their sensitivity to changes in tissue and perform a multimodal study integrated with functional performance parameters (e.g., sprinting biomechanical analyses) that could further increase model accuracy to identify athletes at high risk of HSI. Lastly, the study is limited to elite male athletes. However, we believe that the framework presented in this study could be extended to a more general population irrespective of gender or gender specific (male/ female). This in turn will enable the study of the model differences with respect to gender and different groups (professional vs college athletes).

## Conclusion

In summary, this study investigated radiomics features from prospective MRI scans, prospective strength measures, and injury profile features to predict HSI in professional American football players, some with a prior history of hamstring injuries. The results from this study indicate that AI-derived radiomics features from the hamstring muscles in conjunction with injury profile, morphological characterization, and lower extremity strength variables can predict future HSI. These novel findings merit further additional validation in a larger population and could inform future strategies to identify more clinically meaningful and targetable risk factors to mitigate hamstring strain injury and possibly support a return to sports decision-making following HSI.

## Supporting information

S1 File 3D volume of Hamstring muscle. (MP4)

S2 File 3D volume of Quadricep muscle. (MP4)

S3 File Description of shape and texture based radiomics features. (DOCX)

S4 File 3D volume of feature map for uninjured. (MP4)

S5 File 3D volume of feature map for injured. (MP4)

S6 File Supplementary results. (DOCX)
