# Supplementary material for: Integrating intramuscular fat radiomics with hamstrings-to-quadriceps structure and function ratios to predict future hamstring strain injury
Source: PLOS Digit Health. 2025 Dec 23;4(12):e0001144. doi: 10.1371/journal.pdig.0001144 (PMC12725706; doi:10.1371/journal.pdig.0001144)
Supplement: S3 File — (DOCX) [file pdig.0001144.s003.docx]

**Description of Texture and Shape-based Radiomics Features**

| **Sl. No.** | **Radiomics Features** | **Description** |
| --- | --- | --- |
|  | Shape features (14 features) | These features include the descriptors of the three-dimensional size and shape of the ROI such as elongation, flatness, compactness, length of major and minor axis, volume, surface area, voxel volume, surface volume ratio, sphericity etc. |
|  | First-order statistics (18 features) | These features describe the distribution of voxel intensities within the mask region of image through commonly used metrics such as mean, median, minimum, maximum, energy, entropy, percentiles, range, skewness, kurtosis, variance, deviation etc. |
| 3. | Gray level Co-occurrence matrix (GLCM) (24 features) | GLCM of size $N_{d}\times N_{d}$ describes the second-order joint probability function of an image region enclosed by the mask and is defined as $P(i,j\vert\delta,\alpha)$. The ${(i,j)}^{th}$ element of this matrix represents the number of times the combination of levels $i$  and $j$  occur in two pixels in the image, that are separated by a distance of δ pixels in direction α, and $N_{d}$ is the number of discrete gray level intensities. Metrics such as autocorrelation, average intensity, cluster prominence and contrast are computed. |
| 4. | Gray Level Size Zone matrix (GLSZM) (16 features) | GLSZM quantifies gray level zones in an image. In a gray level size zone matrix $P\left( i,j \right),$ the ${(i,j)}^{th}$ element describes the number of times a gray level zone with gray level $i$ and size j  appears in image. Metrics such as small area emphasis, large area emphasis, intensity variability, Size Zone Variability, Zone Percentage Feature etc. are computed. |
| 5. | Gray Level Run Length Matrix  (GLRLM) (16 features) | GLRLM quantifies gray level runs in an image. A gray level run is defined as the length in number of pixels, of consecutive pixels that have the same gray level value. In a GLRLM $P(i,j\vert\theta)$, the ${(i,j)}^{th}$ element describes the number of times a gray level $i$  appears consecutively $j$ times in the direction specified by θ. |
| 6. | Neighboring Gray Tone Difference Matrix (NGTDM) (5 features) | NGTDM quantifies the difference between a gray value and the average gray value of its neighbours within distance $\delta$. The sum of absolute differences for gray level $i$ is stored in the matrix. |
| 6. | Gray level Dependence matrix (GLDM) (14 features) | GLDM quantifies gray level dependencies in an image. A gray level dependency is defined as the number of connected voxels within distance $\delta$ that are dependent on the center voxel. A neighbouring voxel with gray level $j$ is considered dependent on center voxel with gray level $i$ if $\left\vert i-j \right\vert<\propto$. In a gray level dependence matrix $P(i,j)$ the ${(i,j)}^{th}$ element describes the number of times a voxel with gray level $i$ with j dependent voxels in its neighbourhood appears in image |
