# Supplementary material for: Integrating intramuscular fat radiomics with hamstrings-to-quadriceps structure and function ratios to predict future hamstring strain injury
Source: PLOS Digit Health. 2025 Dec 23;4(12):e0001144. doi: 10.1371/journal.pdig.0001144 (PMC12725706; doi:10.1371/journal.pdig.0001144)
Supplement: S6 File — (DOCX) [file pdig.0001144.s006.docx]

Table A: Results of cross validation for different variables and their combination using KNN

|  | **Model** | **Sensitivity**  **(%)** | **Specificity**  **(%)** | **Accuracy**  **(%)** | **AUC** | **95% CI (AUC)** |
| --- | --- | --- | --- | --- | --- | --- |
| **Radiomics features** | $M_{r}^{H+Q}$ | 59.2 | 67.06 | 63.2 | 0.6578 | 0.65-0.67 |
| **Injury Profile features** | HaOS | 50.36 | 47.6 | 48.96 | 0.4918 | 0.48-0.50 |
|  | Prior HSI | 83.69 | 13.5 | 47.96 | 0.4302 | 0.42-0.44 |
| **Radiomics and injury profile features** | $M_{r+i}^{H+Q}$ | 67.78 | 67.78 | 67.78 | 0.7066 | 0.69-0.72 |
| **Muscle imbalance features** | CSA of HM | 41.11 | 48.31 | 44.78 | 0.4451 | **0.44-0.46** |
|  | CSA of QM | 61.02 | 53.78 | 57.34 | 0.6082 | **0.60-0.62** |
|  | $A_{H:Q}$ | 60.49 | 53.59 | 56.97 | 0.6493 | 0.64-0.66 |
|  | $T_{H:Q}$ | 86.22 | 71.04 | 78.49 | 0.7973 | 0.79-0.81 |
| **Radiomics and**  **Muscle imbalance features** | $M_{r+b}^{H+Q}$ | 69.24 | 67.35 | 68.28 | 0.7275 | 0.72-0.74 |
| **Radiomics, injury profile features and**  **Muscle imbalance features** | $M_{r+b+i}^{H+Q}$ | 75.96 | 70.19 | 73.02 | 0.7804 | 0.77-0.79 |


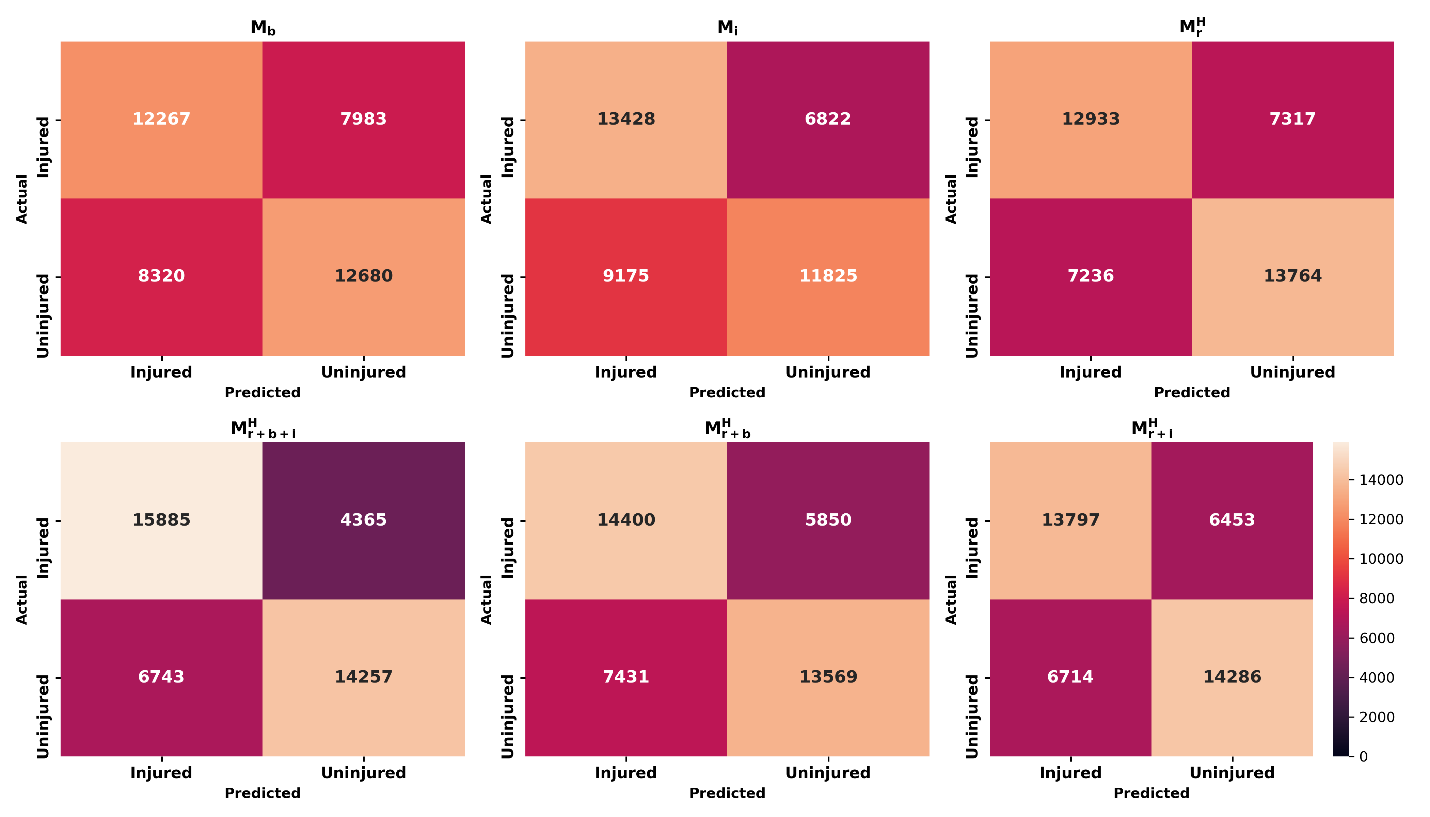
c

Fig A: Confusion matrices for best performing models in each category using knn.

1. **Performance evaluation with other classifiers**

We computed performance of highest performing model in each group (as per Table 2 in revised manuscript) using four different classifiers, namely K-nearest neighbor (KNN), Logistic Regression (LR), Random Forest (RF), Support Vector Machine (SVM) (with rbf kernel). Table A shows the classifier settings. Table B shows performance of different classifiers for the model group. It can be observed that linear classifiers like KNN, LR and SVM (linear) perform better than RF and SVM (rbf kernel).

Classifier settings are:

| Classifier | Settings |
| --- | --- |
| KNN | 11 nearest neighbors, distance-minkowski |
| LR | Penalty-L2, tolerance-0.0001, C=1, max iteration=100 |
| RF | N_estimator=100,criterion=’gini’, minimum samples for split=2, maximum depth=5 |

Table B: Performance evaluation of best model in each group using different classifiers.

| **Model Group** | **Classifier** | **Sensitivity**  **(%)** | **Specificity**  **(%)** | **Accuracy**  **(%)** | **AUC** | **95% CI**  **(AUC** |
| --- | --- | --- | --- | --- | --- | --- |
| **Radiomics features** $\boldsymbol{M}_{\boldsymbol{r}}^{\boldsymbol{H}}$ | **KNN** | **63.87** | **65.54** | **64.72** | **0.6789** | **0.67-0.69** |
|  | LR | 55.24 | 76.55 | 66.09 | 0.6686 | 0.66-0.68 |
|  | RF | 18.58 | 95.9 | 57.94 | 0.767 | 0.76-0.78 |
|  | SVM (rbf) | 36.71 | 90.56 | 64.12 | 0.7201 | 0.71-0.73 |
|  | SVM (Linear) | 56.93 | 73.43 | 65.33 | 0.6787 | 0.67-0.68 |
| **Radiomics features** $\boldsymbol{M}_{\boldsymbol{r}}^{\boldsymbol{Q}}$ | **KNN** | 48.00 | 59.47 | 53.84 | 0.5523 | 0.54-0.56 |
|  | LR | 44.49 | 68.62 | 56.78 | 0.6172 | 0.61-0.63 |
|  | RF | 4.76 | 92.02 | 49.18 | 0.4988 | 0.49-051 |
|  | SVM (rbf) | 23.38 | 81.37 | 52.9 | 0.5346 | 0.52-0.55 |
|  | **SVM (Linear)** | **46.27** | **65.42** | **56.02** | **0.6085** | **0.60-0.61** |
| **Injury profile features** $M_{i}$ | **KNN** | **59.02** | **66.39** | **62.77** | **0.6852** | **0.68-0.69** |
|  | LR | 52.40 | 75.68 | 64.25 | 0.6981 | 0.69-0.71 |
|  | RF | 45.80 | 71.68 | 58.98 | 0.6599 | 0.65-0.66 |
|  | SVM (rbf) | 54.43 | 69.49 | 62.1 | 0.6744 | 0.67-0.68 |
|  | SVM (Linear) | 53.69 | 78.1 | 66.12 | 0.6576 | 0.65-0.67 |
| **Radiomics and injury profile features** $M_{r+i}^{H}$ | **KNN** | **68.13** | **68.03** | **68.08** | **0.7186** | **0.71-0.72** |
|  | LR | 51.78 | 78.97 | 65.62 | 0.6988 | 0.69-0.71 |
|  | RF | 15.78 | 96.57 | 56.91 | 0.7636 | 0.76-0.77 |
|  | SVM (rbf) | 32.53 | 93.01 | 63.32 | 0.7363 | 0.73-0.75 |
|  | SVM (Linear) | 52.84 | 77.26 | 65.27 | 0.7109 | 0.70-0.72 |
| **Radiomics and injury profile features** $M_{r+i}^{Q}$ | **KNN** | **54.71** | **63.06** | **58.96** | **0.5987** | **0.59-0.61** |
|  | LR | 42.71 | 75 | 59.15 | 0.6529 | 0.64-0.66 |
|  | RF | 3.07 | 92.51 | 48.6 | 0.4921 | 0.48-0.50 |
|  | SVM (rbf) | 19.02 | 85.16 | 52.69 | 0.5402 | 0.53-0.55 |
|  | SVM (Linear) | 43.73 | 72.52 | 58.39 | 0.6412 | 0.63-0.65 |
| **Muscle imbalance features** $M_{b}$ | **KNN** | **60.58** | **60.38** | **60.48** | **0.6419** | **0.63-0.65** |
|  | LR | 67.29 | 69.5 | 68.42 | 0.79 | 0.78-0.79 |
|  | RF | 32.18 | 90.67 | 61.96 | 0.743 | 0.73-0.75 |
|  | SVM (rbf) | 54.8 | 67.59 | 61.31 | 0.6534 | 0.64-0.66 |
|  | SVM (Linear) | 68.93 | 67.06 | 67.98 | 0.7869 | 0.78-0.79 |
| **Radiomics, muscle imbalance**  **Features** $M_{r+b}^{H}$ | **KNN** | **71.11** | **64.61** | **67.8** | **0.7358** | **0.73-0.75** |
|  | LR | 53.29 | 80.25 | 67.02 | 0.72 | 0.71-0.73 |
|  | RF | 19.73 | 97.54 | 59.35 | 0.8005 | 0.79-0.81 |
|  | SVM (rbf) | 34.31 | 92.69 | 64.03 | 0.7515 | 0.74-0.76 |
|  | SVM (Linear) | 52.67 | 78.42 | 65.78 | 0.7161 | 0.70-0.73 |
| **Radiomics, muscle imbalance**  **Features** $M_{r+b}^{Q}$ | **KNN** | **66.13** | **64.84** | **65.47** | **0.6981** | **0.69-0.71** |
|  | LR | 58.36 | 81.1 | 69.93 | 0.7988 | 0.79-0.81 |
|  | RF | 11.64 | 96.3 | 54.74 | 0.7145 | 0.71-0.73 |
|  | SVM (rbf) | 25.11 | 90.73 | 58.52 | 0.7107 | 0.70-0.72 |
|  | **SVM (Linear)** | 57.69 | 80.01 | 69.05 | 0.7799 | **0.77-0.79** |
| **Radiomics, muscle imbalance, and injury profile features** $M_{r+b+i}^{H}$ | **KNN** | **78.44** | **67.89** | **73.07** | **0.7879** | **0.78-0.80** |
|  | LR | 54.53 | 82.14 | 68.59 | 0.7589 | 0.75-0.77 |
|  | RF | 17.42 | 97.37 | 58.12 | 0.7983 | 0.79-0.80 |
|  | SVM (rbf) | 30.76 | 94.08 | 62.99 | 0.7741 | 0.77-0.78 |
|  | SVM (Linear) | 50.76 | 81.1 | 66.21 | 0.7514 | 0.74-0.76 |
| **Radiomics, muscle imbalance, and injury profile features** $M_{r+b+i}^{Q}$ | **KNN** | **63.60** | **67.61** | **65.64** | **0.6932** | **0.68-0.70** |
|  | LR | 55.24 | 83.39 | 69.57 | 0.8192 | 0.81-0.82 |
|  | RF | 8.13 | 96.4 | 53.07 | 0.7064 | **0.69-0.72** |
|  | SVM (rbf) | 24.71 | 92.63 | 59.29 | 0.713 | 0.70-072 |
|  | SVM (Linear) | 53.42 | 84.03 | 69.01 | 0.8095 | 0.80-0.82 |

Table C: False positive and false negative rate of best performing models

| **Model** | **False Negative Rate (%)** | **False Positive Rate (%)** |
| --- | --- | --- |
| $M_{b}$ | 39.42 | 39.62 |
| $M_{i}$ | 33.69 | 43.69 |
| $\boldsymbol{M}_{\boldsymbol{r}}^{H}$ | 36.13 | 34.46 |
| $\boldsymbol{M}_{\boldsymbol{r}+\boldsymbol{b}+\boldsymbol{i}}^{H}$ | 21.56 | 32.11 |
| $\boldsymbol{M}_{\boldsymbol{r}+\boldsymbol{b}}^{H}$ | 28.89 | 35.39 |
| $\boldsymbol{M}_{\boldsymbol{r}+\boldsymbol{i}}^{H}$ | 31.87 | 31.97 |

**Note:** One subject in the uninjured group was missing the segmentation mask for the left Vastus Lateralis muscle.
